# Jasmonates: News on Occurrence, Biosynthesis, Metabolism and Action of an Ancient Group of Signaling Compounds

**DOI:** 10.3390/ijms19092539

**Published:** 2018-08-27

**Authors:** Claus Wasternack, Miroslav Strnad

**Affiliations:** 1 Department of Molecular Signal Processing, Leibniz Institute of Plant Biochemistry, Weinberg 3, D-06120 Halle (Saale), Germany; 2 Laboratory of Growth Regulators, Institute of Experimental Botany AS CR & Palacký University, Šlechtitelů 11, CZ-78371 Olomouc, Czech Republic; miroslav.strnad@upol.cz

**Keywords:** occurrence, Jasmonic acid (JA) metabolites, JA biosynthetic enzymes, JA bypass, active JA compounds, JA signaling, transcription factors

## Abstract

Jasmonic acid (JA) and its related derivatives are ubiquitously occurring compounds of land plants acting in numerous stress responses and development. Recent studies on evolution of JA and other oxylipins indicated conserved biosynthesis. JA formation is initiated by oxygenation of α-linolenic acid (α-LeA, 18:3) or 16:3 fatty acid of chloroplast membranes leading to 12-oxo-phytodienoic acid (OPDA) as intermediate compound, but in *Marchantia* *polymorpha* and *Physcomitrella* *patens*, OPDA and some of its derivatives are final products active in a conserved signaling pathway. JA formation and its metabolic conversion take place in chloroplasts, peroxisomes and cytosol, respectively. Metabolites of JA are formed in 12 different pathways leading to active, inactive and partially active compounds. The isoleucine conjugate of JA (JA-Ile) is the ligand of the receptor component COI1 in vascular plants, whereas in the bryophyte *M. polymorpha* COI1 perceives an OPDA derivative indicating its functionally conserved activity. JA-induced gene expressions in the numerous biotic and abiotic stress responses and development are initiated in a well-studied complex regulation by homeostasis of transcription factors functioning as repressors and activators.

## 1. Introduction

Plants are sessile, but have to adapt to changes of abiotic factors such as light, salt, nutrient deficiency, water deficit or cold. Additionally, biotic interactions with pathogens, herbivores, nematodes or symbiotic microorganisms occur. Jasmonic acid (JA) and its isoleucine conjugate (JA-Ile) are among the most important signals of these different stress responses and are active in root growth, seed germination, stamen development or senescence.

JA and its derivatives originate from lipids of chloroplast membranes, preferentially α–linolenic acid (α-LeA). Synthesis takes place by oxygenation of α-LeA in one of the different branches of the so-called lipoxygenase (LOX) pathway.

Beside formation of JA via octadecanoids, these branches lead to leaf aldehydes and alcohols, as well as divinyl ether-, epoxy hydroxy-, hydroxy-, and keto-polyunsaturated fatty acids (PUFAs) [1]. Oxygenation by LOXs takes place at carbon atom 9 (9-LOX) or carbon atom 13 (13-LOX). 13-LOX are active with esterified or free fatty acids. In case of Arabidopsis, galactolipids contain esterified OPDA at different positions, collectively called arabidopsides. They are prominent examples for esterified substrates of 13-LOXs. The JA branch within the LOX pathway requires 13-LOXs and the subsequent steps of JA formation have been elucidated by Vick and Zimmermann in 1984 (reviewed in [2] cf. Part 3).

Many aspects of JA/JA-Ile, addressed briefly in the following overview, on occurrence, biosynthesis, metabolism, perception, and signaling have been permanently reviewed. Examples are [1,3,4,5,6,7,8,9].

## 2. Occurrence of Jasmonic Acid (JA) Compounds

JA and components of its biosynthesis and signaling pathway, respectively, do not occur in yeast, in animal and human tissues, but in some prokaryotes, some lower and all higher plant species [10,11,12]. The first detected JA compound was the methyl ester of JA (JA-Me) found in the odor of flowering plants [13] and in the culture medium of the fungus *Lasiodiplodia theobromae* (synonym: *Botryodiplodia theobromae*) [14]. Later on, specific stereo-isomeric forms of JA such as (+)-*7-iso*-JA and its derivatives were detected in *L. theobromae* [15,16]. Several JA esters, so-called lasiojasmonates, were isolated from different Lasiodiplodia species [17]. In the grapevine pathogen *L. mediterannea* sp. the JA furanoyl ester LasA was detected, can be transformed into the bioactive JA-Ile and seems to function as an inactive JA pool [18]. Currently, *L. theobromae* is studied by a project for genome sequencing (http://genome.jgi-psf.org/pages/fungi-1000-projects.jsf) which will help to explain the already detected occurrence of numerous JA derivatives including hydroxylated and conjugated JA derivatives (reviewed by [1,19]). Numerous JA derivatives occur also in the fungus *Fusarium oxysporum* [20] with the allene oxide 12,13(*S*)-epoxy-octadecatrienoic acid and OPDA as key intermediates [21], which indicates similarity of JA formation between fungi and plants. The JA precursor OPDA, but not JA, have been detected in *Marchantia polymorpha* [22], in the moss *Physcomitrella patens* [23] and in one of the oldest vascular plants, the spikemoss *Selaginella martensii* [24], whereas in *S. moellendorffii* OPDA and JA were detected [25]. Even though no JA occurs in *M. polymorpha*, essential components of JA signaling have been found suggesting occurrence of another ligand than JA-Ile [11]. Indeed, two isomeric forms of the JA-Ile precursor dinor-OPDA, dinor-*cis*-OPDA and dinor-*iso*-OPDA, have been identified recently, suggesting co-evolution in JA-Ile perception and ligand/receptor specificity [26] (cf. Parts 7 and 9.3). In contrast, orthologues of genes encoding enzymes of JA biosynthesis, perception and signaling could not be found in the Chara genome [10]. Continuous improvement of analytical tools may contribute to proving or disproving some of the preliminary results available for lower organisms [27,28].

In higher land plants, JA compounds occur ubiquitously [29]. Even the conjugate of OPDA with isoleucine has been found in flowering *A. thaliana* [30] and showed biological activity [31]. Its formation from the Ile conjugate of α-LeA was shown recently by in vitro enzymatic methods [32].

## 3. Biosynthesis of JA

**Figure 1:**
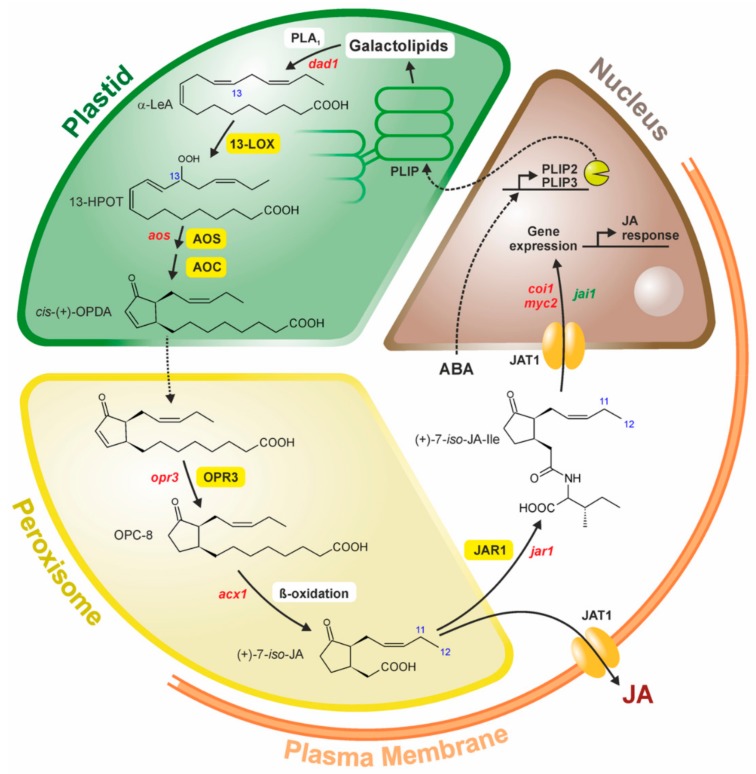
Scheme on Jasmonic Acid/Jasmonic Acid Isoleucine Conjugate (JA/JA-Ile) biosynthesis and action in four different compartments of a plant cell. Upon release of α-linolenic acid by the A_1_-type lipases (PLIP, Plastid Lipase; PLA_1_, Phospholipase A1 of flowers) from galactolipids, *cis*-(+)-12-Oxophytodienoic Acid (*cis*-(+)-OPDA) is formed in plastids by a 13-LOX, AOS and AOC. Following transport into peroxisomes, OPDA is reduced by OPDA reductase3 (OPR3) und undergoes ß-oxidation of the carboxylic acid side chain to JA (shown as the initially formed right configuration (+)-*7-iso*-JA). Upon release into the cytosol, JA is conjugated with amino acids by JAR1 or exported by a JA-transporter JAT1. The same transporter allows import of JA-Ile into the nucleus, where JA-Ile perception and JA-Il-induced gene expression takes place (for details and references cf. Part 3). Mutants known for Arabidopsis (red) or tomato (green) are indicated.

The pathway of JA biosynthesis has been elucidated as a route of oxygenation of lipid-derived fatty acids with OPDA as intermediate [33] (Figure 1).

Recently, a bypass via didehydro-JA has been identified (cf. [34]) and is discussed below. All genes of enzymes active in JA biosynthesis were cloned from numerous species, and biochemical properties, subcellular location, crystal structure, mechanism, and regulation of these enzymes have been identified. These aspects have been permanently reviewed (for details, see References [1,3,7,8,35,36]). Here, we will focus on new aspects arising in recent years.

### 3.1. Galactolipases Active in JA Biosynthesis

Initially, the flower-specific protein DEFECTIVE IN ANTHER DEHISCENCE 1 (DAD1), a phospholipase A1 (PLA_1_) was identified to be absolutely required for JA formation of flowers [37], but involvement of a PLA1 homolog of leaves were controversially discussed (cf. review in Reference [7]). Preferentially, there is a pathway- and stimulus-specific action of lipases in JA biosynthesis.

An interesting link between biosynthesis of lipid digalactosyldiacylglycerol (DGDG) and JA biosynthesis was described [38]. The mutant *DGDG synthase1*, *dgd1*, has a reduced photosynthesis, altered chloroplast morphology, strongly reduced DGDG content, as well as up-regulation of a 13-LOX and phospholipase A-Iγ3 which is preferential active with mono galactosyldiacylglycerol (MGDG), the major substrate for JA biosynthesis. The mutant has elevated levels of JA, JA-Ile, OPDA, and arabidopsides which seems to be triggered upon increased ratio of MGDG to DGDG [38] leading to the growth phenotype of *dgd1* plants. Obviously, there is a complex link between MGDG and DGDG homeostasis, JA formation, growth inhibition, chloroplast shape, phosphate starvation and freezing tolerance [39].

More recently, new data has clarified a long-term puzzle in which the numerous lipases, preferentially galactolipases, are involved in JA biosynthesis: Upon identification of the PLASTID LIPASE (PLIP1) active in export of acyl groups from plastids for seed oil biosynthesis [40], the homologs PLIP2 and PLIP3 were shown to have a glycerolipase A1 activity leading to release of MGDG (in case of PLIP2) and phosphatidylglycerol (in case of PLIP3). Their expression is ABA- and COI1-dependent, and the triple mutant *plip1plip2plip3* is unable to form JA [41] (Figure 1). This is a mechanistic explanation for the stimulatory effect of ABA on JA formation in numerous stress responses, a cross-talk between ABA and JA which has been known for more than two decades [2,42].

### 3.2. 13-Lipoxygenase (LOX)

13-LOXs are well-studied by cloning and enzymatic characterization for several species. In tomato, two of six LOXs, TomloxC and TomloxD, are located within the chloroplast. TomloxC is involved in C5 flavor volatile formation without a role in defense [43]. TomloxD is affected in the mutant *spr8* and is involved in JA formation and defense against herbivores [44]. In the mutant *spr8*, the catalytic domain has a point mutation. In poplar there is a large *LOX* gene family of 20 members [45]. In rice, *OsLOX2* and *OsLOX5* are specifically suppressed by microRNA319 which takes place via suppression of the microRNA319-target *OsTCP21* (TEOSINTE BRANCHED/CYCLOPEDEA/PROLIFERATING CELL FACTOR) [46]. Thus, the *OsLOX2, OsLOX5* and microRNA319 are key components of rice defense upon *Magnaporte oryzae* infection [46]. In Arabidopsis there are four 13-LOXs (AtLOX2, AtLOX3, AtLOX4, AtLOX6) and two 9-LOXs (AtLOX1, AtLOX5). The 9-LOXs seem to be involved in formation cell death by synthesis of death acids [47]. All 13-LOXs of Arabidopsis are involved in JA formation with partially specific activities in wounding, lipid peroxidation, wound response in vascular tissues, natural and dark-induced senescence, fertility and flower development.

There is a transcriptional and translational control of 13-LOXs in these processes, reviewed in Reference [3]. AtLOX6 is essential for JA formation in roots in response to biotic and abiotic stress [48], whereas AtLOX3 functions in salinity stress [49] and AtLOX4 is involved defense reactions against root-knot nematodes [50]. A hierarchy of action of the four 13-LOXs has been detected for Arabidopsis [51]. AtLOX2 and AtLOX6 are active upstream of AtLOX3 and AtLOX4 in several responses. AtLOX2 is developmentally regulated and target of AtTCPs which are targets of miRNA319 [52]. AtLOX2 is involved in a translational control due its interaction with the eukaryotic initiation factor 4E [53] (for details cf. review in Reference [3]). Interestingly, antisense lines of a rice 9-LOX1 exhibited increased JA levels and JA-mediated responses against chewing and phloem-feeding herbivores, indicating a cross-talk between 13-LOX and 9-LOX [54].

The formation of numerous JA derivatives in the fungus *F. oxysporum* [20] can now be explained by multifunctional properties of one of the two LOXs. Whereas the initially described LOX was a 13*R*-Mn-FoxLOX, the second FoxLOX was identified as regular 13-LOX with dioxygenase and hydroperoxidase activity [55]. 

Crystal structure of several LOXs has been elucidated and allowed an improved mechanistic explanation of their catalytic mechanism [56,57]. The substrates can be alternatively positioned due to the conserved core thereby allowing formation of different products [56]. In case of LOX1 of the cyanobacterium *Cyanothece* sp. PCC 8801 an α-helical extension attributes to substrate acquisition directly from the membrane [57]. Mechanistic details of LOXs were discussed in a recent review [1].

### 3.3. Allene Oxide Synthase (AOS)

The *CYP74* gene family consists of AOS, and the hydroperoxide lyase (HPL) as well as divinylether synthase (DES) [58]. Previous reviews covered cloning, characterization, mechanism and function of AOS in stress responses and development [36,59]. Beside the single copy gene of Arabidopsis [60], many plants carry *AOS* gene families. The AOS from Arabidopsis has been crystalized in free and complexed form with the substrate which led to mechanistic discussion on AOS versus HPL activity and on evolutionary origin of AOS [61].

Based on characterization of the two AOSs from *M. polymorpha*, multiple evolution of CYP74 enzymes prior to divergence of the flowering plants has been discussed [62]. This corresponds to data for the two AOS of *P. patens*, where the PpAOS1 and PpHPL were inter-conversable by a single amino acid exchange [63]. Additionally, in higher plant species such as *Medicago truncatula*, *Cucumis melo*, or *Glycine max* conversion of HPLs into AOSs can be performed by site-directed mutagenesis [64]. In *F. oxysprum* containing allene oxide and OPDA as key intermediates similar to plants [21], the AOS occurs as fusion protein with 8- and 9-dioxygenases activity [65].

In many plant species, AOSs are specific for either 9- or 13-hydroperoxide derivatives. In rice AOS1 [66] and barley AOS1 [67], however, dual positional substrate specificity has been detected. An interesting mutant was found upon screening of a rice library on constitutive *AOS* expression. This mutant, called *cea62*, is affected in OsHPL3, and showed elevated JA levels which suggests a link between the AOS and the HPL branch [68]. In contrast, low levels of JA occurring in *precious* (*pre*) rice mutant due to a mutated OsAOS1, attribute to a long leaf phenotype [69] suggesting that JA is a negative regulator in vegetative development [69]. A new group of oxylipins which are generated via the AOS pathway, has been identified in cereals [70]. These so-called graminoxines are formed from compounds such as (9*Z*,11*E*,13*S*)-13-hydroperoxy-9,11-octadecadienoic acid via hydrolysis of a short lived AOS-generated cyclopentanone to (4*Z*)-2-pentyl-4-tridecene-1, 13-dienoic acid (graminoxin A1), which has a carboxy function at the side chain.

### 3.4. Allene Oxide Cyclase (AOC)

*AOCs* occur in small gene families which have been characterized from several plant species (cf. reviews of [36,71]. Crystal structures are available for AtAOC2 [72] and PpAOC1 and PpAOC2 [73].

The recombinant AOC of the liverwort *M. polymorpha*, where no JA occur, has similar properties like that of flowering plants [22]. Since *M. polymorpha* contains essential components of JA signaling [11], signaling properties of OPDA or a so-far-unidentified derivative may occur [22] (cf. Parts 7 and 9.3) [22]. Generally, there is no elevated JA level upon over-expression of *AOS* or *AOC*, since the required substrate α-LeA is generated only upon external stimuli such as wounding [74]. Exceptions are *Salvia miltiorrhiza* and wheat, where constitutive over-expression of *AOC* led to elevated JA levels and JA responses in the transgenic plants [75,76]. In rice, the mutant *coleoptile photomorphogenesis 2* (*cpm2)* and *hebiba* are affected in the AOC. Both mutants exhibited deficiency in OPDA, increased salt tolerance and ROS-scavenging activity as well as altered defense against the blast fungus *M. oryzae* [77,78]. Expression data and a proteomic analysis of the rice mutant *cpm2* suggest a negative regulatory role of JA in drought tolerance [79]. In Arabidopsis, the four members of the *AOC* gene family are expressed in tissue- and organ-specific manner [80]. An activity control of AOCs via heteromerization was found in vitro and in vivo and was functionally proved by site-directed mutagenesis [81]. OPDA is the final product of the first half of JA formation localized within plastids. For research purpose, there is permanent demand for OPDA. Now, OPDA can be synthesized by covalently immobilized recombinant rice AOS-1, together with bound soybean LOX and rice AOC, which are able to form OPDA from α–LeA up to more than 80% yield [82] or by enzymatic multi-step one-pot synthesis using all three enzymes from *A. thaliana* and α–LeA as substrate [83].

### 3.5. OPDA Reductase (OPR3)

The second half of JA biosynthesis takes place in peroxisomes upon transport of OPDA across two membranes (Figure 1). A transporter of chloroplast envelope membranes has not been identified so far. A putative transport activity might occur by the intrinsic acyl-CoA thioesterase activity of a fatty acid-transporting peroxisomal ATP binding cassette transporter [84]. More clear evidence is given for the import of OPDA into peroxisomes by peroxisomal ABC transporter1 (PXA1, also called COMATOSE, CTS1) or by an anion trapping [85]. In the peroxisome, reduction of OPDA takes place by OPRs which occur in small *OPR* gene families. Cloning, as well as characterization including crystallization of OPRs have been reviewed [3,36,59]. Within the *OPR* gene families, only distinct members are involved in JA biosynthesis. Among the 10 members of rice, only OsOPR7 was identified as JA-forming enzyme [86]. In Arabidopsis, among six AtOPRs, AtOPR3 was exclusively thought to be involved in JA formation indicated by the dominant formation of the *3R,7S* stereoisomer. This corresponds to the residual amount of the unnatural stereoisomer *3R,7R* form wounded *opr3-1* plants. The first identified mutant, *opr3-1*, is JA-deficient but accumulates OPDA upon wounding [87,88]. For more than 10 years, *opr3-1* plants were used to distinguish between OPDA- and JA-specific responses which were found increasingly detected in Arabidopsis (cf. below). *opr3-1* plants are male sterile due to the essential role of JA in stamen development [35,89,90]. Later on, however, conditional JA formation was shown due to intronic T-DNA insertion in the *opr3-1* mutant line [91] (cf. Part 4).

Interestingly, a novel function of *AtOPR3* was shown under *p* deficiency, where root tip growth is suppressed by *AtOPR3* at transcriptional level [92].

### 3.6. β-Oxidation of the Carboxylic Acid Side Chain (ACX, MFP, KAT)

The carboxylic acid side chain of JA is shortened by the fatty acid β-oxidation machinery which is similar to the final steps in auxin biosynthesis [93]. The β-oxidation of OPDA is shortened in JA formation as shown by labeling experiments [94] as well as by detection of involved enzymes such as acyl-CoA oxidase (ACX) [95,96], multifunctional protein (MFP) [97], L-3-ketoacyl CoA thiolase (KAT) [98] and 4-coumarate: CoA ligase-like enzymes [99]. Further data were found by diminished JA formation in *pex* mutants which are affected in components of the peroxisomal import complex [93].

Normal inflorescence development requires JA. The evolutionary conserved KAT1 and KAT5 are required for Arabidopsis inflorescence development [100] corresponding to the flower inflorescence phenotype of the *aim1* mutant affected in the MFP protein [97].

## 4. The Bypass in JA Formation-the COI1-Independent and OPR3-Independent Route

**Figure 2:**
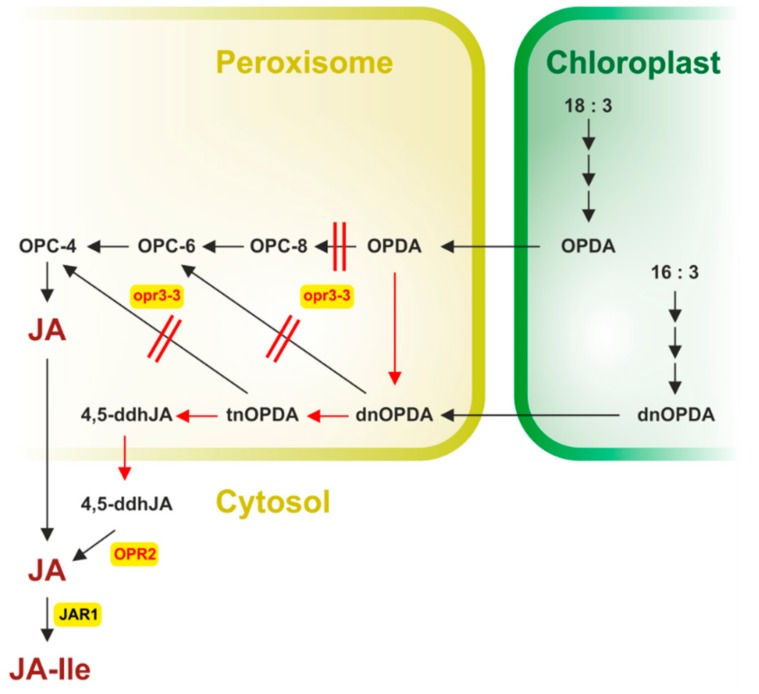
The bypath in JA biosynthesis. OPDA and dnOPDA are formed in chloroplasts and converted to JA and 4,5-ddh-JA, respectively, in peroxisomes. In *opr3-3* plants, peroxisomal OPDA is metabolized to dinor-OPDA (dnOPDA), tetranor-OPDA (tnOPDA) and 4,5-didehydro-JA (4,5-ddh-JA), which is reduced to JA by OPR2 after release into the cytosol. Black arrows show the canonical pathway, red arrows highlight the new pathway identified in *opr3-3* plants. Red parallel lines indicate block in the conversion of OPDA and dnOPDA within the *opr3-3* mutant. The newly identified property of OPR2 in *opr3-3* is marked in red (redrawn based on Reference [102] with permission).

As described above, the initially identified pathway of JA biosynthesis consists in fatty acid oxygenation, its cyclization, reduction of the cyclopentenone ring and shortening of the carboxylic acid side chain (Figure 1). A crucial step is the OPR3-catalyzed step which leads to cyclopentanone compounds. The first identified mutant allele, *opr3-1*, was a conditional mutant able to form *OPR3* mRNA and JA under few conditions [91]. Now, a new mutant allele, *opr3-3*, has been identified which shows complete absence of OPR3 activity [34]. Therefore, complete JA-deficiency was expected. Detailed genetic, biochemical and analytical analyses, however, led to identification of a bypath of JA formation via dinor-OPDA and 4,5-ddh-JA (Figure 2) [34]. This has been discussed recently in two simultaneously published commentaries, References [101,102].

*opr3-3* plants accumulate upon wounding 4,5-ddh-JA and JA-Ile, but not 4,5-ddh-JA-Ile suggesting a β-oxidation of the carboxylic acid side chain of OPDA to dinor-OPDA (Figure 2).

JA-like activity of 4,5-ddh-JA was found in typical JA responses such as the root growth inhibition for the wild type and *opr3-3* plants but not in *coi1-30* and *jar1-1* mutant plants, which indicates requirement of an active COI1 and JAR1 protein. Finally, biochemical and genetic data, as well as feeding experiments with deuterated α-LeA followed by quantification of deuterated 4,5-ddh-JA, deuterated JA and deuterated JA-Ile, showed reduction of the cyclopentenone ring of 4,5-ddh-JA by OPR2 to JA followed by conjugation to JA-Ile (Figure 2). Previously, OPR2 and OPR1 were thought to be active in the reduction of *α,β*-unsaturated carbonyls (conjugated enones) of various compounds [59]. Now, the new biological activity of OPR2 suggests evolutionary different pathways depending on occurrence of different OPRs. Indeed, OPR3 orthologues are absent in lower plants such as bryophyta corresponding to the absence of JA in these plants (cf. above) but carry *OPR2*-like genes [12]. This corresponds to the preferential occurrence of the OPR3-dependent route in vascular plants [12].

## 5. Regulation of JA Biosynthesis

External stimuli lead to release of α-LeA from chloroplast membranes which is required as substrate in JA biosynthesis. The expression of JA-biosynthesis genes is feedback regulated by JA, but this assumption has been criticized in Reference [103]. Furthermore, tissue specificity is of regulatory role in JA biosynthesis. All of these aspects have been reviewed in References [3,7,59]. Additional factors are (i) concurrent activity between the AOS and HPL branch, (ii) negative regulation by JAZs, (iii) heteromerization (e.g., of AOCs of Arabidopsis) [81] (iv) Ca^2+^ signaling, and the mitogen-activated protein kinase cascade (see the review in Reference [3]). Several environmental stimuli lead to up-regulation JA-biosynthesis genes. Wounding is the most prominent example (cf. reviews in References [3,59]), and negative regulation has been shown, e.g., of LOX-F, AOS and AOC by over-expression of the blue light photoreceptor cryptochrome1 [104]. Other putative levels of regulation might be the dimerization of OPR3. Regulation by Ca^2+^ via the 13-LOX activity is suggested due to the identification of the so-called *fou2* mutant affected in the above-mentioned TCP protein [105]. TCPs are among the mi319-controlled transcription factors [52]. 

## 6. Arabidopsides

18:2 and 18:3 fatty acids and their LOX-generated 9- and 13-hydroperoxides occur in free and esterified form in many plant species [106,107]. OPDA and dinor-OPDA occur esterified in large quantities in the *sn-1* or *sn-2* position of the galactolipids MGDG and DGDG of Arabidopsis and these compounds were called arabidopside A-F [108].

**Figure 3:**
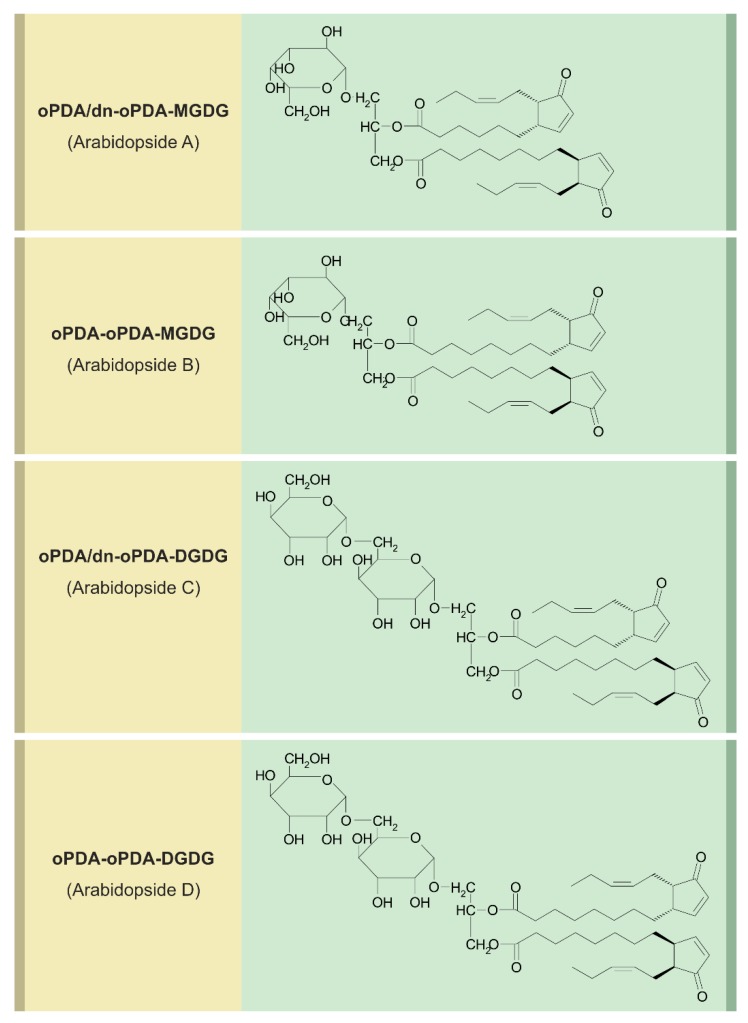
Structures of Arabidopside A, B, C, and D.

In Figure 3 arabidopside A–D are shown as examples.

Initially, the major fraction of OPDA was detected esterified in arabidopsides, and OPDA was shown to be released upon wounding, leading to a dramatic burst in free OPDA and JA [109]. The link between de novo synthesized JA and JA formed upon release of OPDA from arabidopsides is still a matter of discussion [110]. Later on, their involvement in the hypersensitive response as well as in abiotic stress responses led to an ongoing discussion on putative role of arabidopsides [111,112].

AtLOX2 is preferentially responsible for the large amount of JA accumulating proximal to the wound side, for the local wound-induced formation of arabidopsides and can oxidize bound fatty acids [16,113]. This is followed by AOS activity, active on its bound substrate [60,114]. Arabidopsides accumulate also upon abiotic stress, e.g., under natural conditions by stress of copper chloride in the wild crucifer *Erucastrum canarinese* [115]. This formation of arabidopsides takes with a concomitant formation of phytoalexins. Accumulation of arabidopsides occurs differentially in different ecotypes [116]. This is significantly linked to expression of *AtHPL1* which has influence on AOS activity: High accumulation of arabidopsides are negatively correlated to expression of the *AtHPL* which competes with AOS on bound LOX2-generated hydroperoxides [116]. Even some reactions in arabidopside formation were initially assumed to occur spontaneously, but now all steps known so far are proved to be catalyzed by any enzymes [114]. Acylated MDGDs, which occur in parallel to arabidopsides, were detected in many plant species [117]. OPDA is frequently incorporated into acylated MDGDs. Mutants affected in the acylating enzyme exhibit complete loss of OPDA-containing acyl-MDGD, but any biological role of acyl-MDGDs is not known so far [117]. Elevated levels of arabidopsides were found also in the mutant *dgd1* as described in chapter 3.1. Initially, Arabidopsides were detected exclusively, in Arabidopsis [118]. More recently, galactolipids with esterified dihydroJA, dihydroOPDA and OPDA have been identified in leaves of *Cirsium arvense* upon infection with the endophyte *Chaetomium cochlioides* [119]. Such an endophyte-based defense reaction including arabidopside formation may be attributed to systemic signaling and “priming” [119], which are well-known events in the wound response and response to bacterial infection. Arabidopside A, C, and D were also detected in wild crucifers such as *Erucastrum canariense* [115].

## 7. Evolution of JA Biosynthesis

As briefly mentioned above, JA compounds—including the precursor OPDA—occur ubiquitously in higher plants, and have been detected in lower organisms such as fungi, prokaryotes, some cyanobacteria [12,120], green algae and bryophyta, indicating occurrence of functionally active enzymes in JA biosynthesis. Indeed, in prokaryotes and cyanobacteria, homologs of JA biosynthetic enzymes and signaling components have been detected [12]. In the liverwort *M. polymorpha*, the earliest diverging clade of land plants [121], enzymes of OPDA synthesis such as AOC have been found [22], but no homologs were found of downstream steps in JA biosynthesis corresponding to the already mentioned lack of JA in *M. polymorpha*. As mentioned in Part 2, this is similar to *P. patens*, where two AOCs have been characterized and crystallized [23,73].

Genome analysis of *P. patens* [122] and of *M. polymorpha* [11] led to evolutionary insights on JA biosynthesis and signaling. While in these species OPDA occurs, OPR3 required for JA synthesis is missing, but components of JA perception and signaling such as COI1, co-receptors, JAZ repressors, MYC transcription factors as well as NINJA adaptor and the TOPLESS (TPL) co-repressor have been found [11], suggesting a new ligand within a conserved signaling pathway. Dinor-*cis*-OPDA and dinor-*iso*-OPDA, have been identified recently as such new ligands [26] (cf. Part 2 and Part 9). Genome-wide similarity search showed occurrence of all homologs of JA biosynthesis components in major lineages of land plants, whereas only some were found in cyanobacteria, chlorophytes, rhodophytes, and glaucophytes [12]. No homologs of JA-Ile biosynthesis components were found in the red algae *Chondrus crispus* [123]. A comparative genomic and phylogenetic approach on the origin of the nine major plant hormone biosynthesis and signaling pathways revealed four different groups: (i) cytokinin, auxin and strigolactone pathways go back to the charophyte lineage, (ii) abscisic acid, JA and salicylic acid pathways have a common origin in land plants, (iii) gibberellin pathways evolved after branch-off of bryophytes from land plants, (iv) brassinosteroid pathways were created with the appearance of angiosperms presumably after the division of gymnosperms and angiosperms, and finally (v) ethylene pathways seem to have emerged after the first angiosperms [124]. Further aspects of hormone biosynthesis, signaling, development and phylogeny of plants have been reviewed [11,12,124,125].

JA compounds and components of JA-Ile biosynthesis occur in prokaryotes including pathogenic bacteria [12]. Some of them—such as *Pseudomonas syringae*—form the phytotoxin coronatine which is a molecular mimic of JA-Ile action in higher plants. Coronatine, however, does not occur in higher plants. It underpins successfully the bacterial pathogenicity, e.g., in case of infection by *Pseudomonas syringae*, and induces JA signaling and action due to its high activity compared JA [126]. Obviously, in bacteria, coronatine has been evolved next to JA in a type of co-evolution to achieve higher activity [127]. Based on the diverse action of the bacterial-derived coronatine in plant growth and development, studies on structure-activity relationship resulted in the fact that coronatine has been regarded as an attractive herbicidal lead compound [128]. It is, however, difficult to imagine a specific herbicidal action of coronatine regarding the numerous processes affected by jasmonates. 

## 8. Homeostasis Among JA Compounds by Metabolism

**Figure 4:**
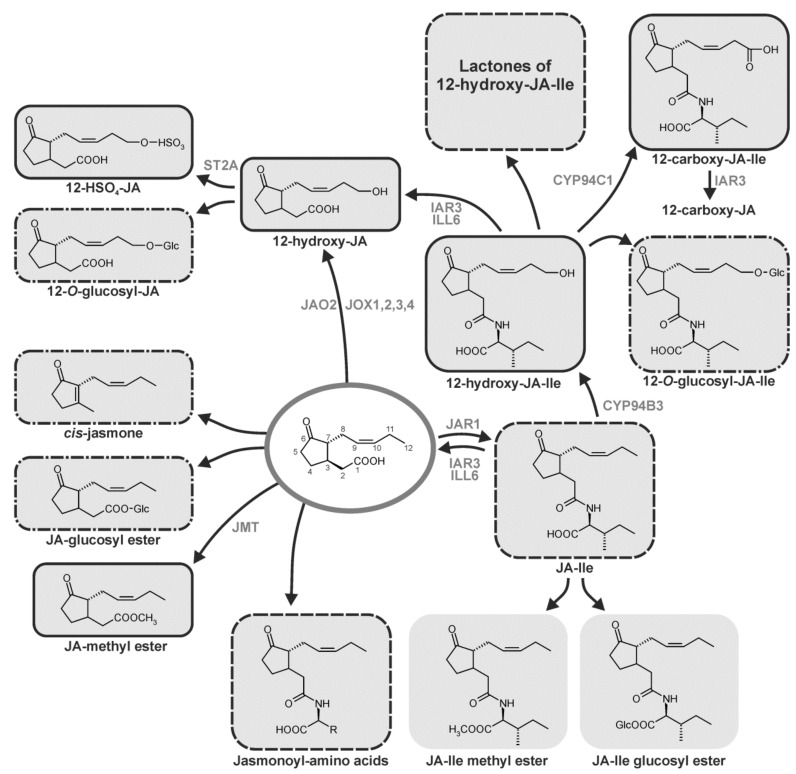
Metabolism of JA. Methylation to JA-methyl ester, JA glucosyl ester formation, decarboxylation to *cis*-jasmone, hydroxylation to 12-hydroxyJA, sulfation of 12-hydroxyJA, *O*-glucosylation of 12-hydroxyJA to 12-*O*-glucosyl-JA, conjugation of JA with amino acids, preferentially isoleucine to give JA-Ile, methylation to JA-Ile methyl ester, 12-hydroxylation of JA-Ile, carboxylation of 12-OH-JA-Ile, *O*-glucosylation of JA-Ile to 12-O-glucosyl-JA-Ile, and formation of JA-Ile glucosyl ester are indicated. Known involved enzymes are JAO2, jasmonic acid oxidase2, JOX1,2,3,4, jasmonate induced oxidase 1,2,3,4, JAR1, jasmonoyl-isoleucine synthetase, JA-Ile-12-hydroxylase CYP94B3, 12-OH-JA-Ile carboxylase CYP94C1, amidohydrolases IAR3 and ILL6, JMT, JA methyl transferase, ST2a, 12-OH-JA sulfotransferase. The lactone of 12-OH-JA-Ile was added even though detection for plants is missing so far. Biologically inactive compounds are outlined with solid lines (▬▬▬), partially active compounds are outlined with dashed and dotted lines (▬ **^.^**▬ **^.^**▬) and active compounds are outlined with dotted lines (-----), the biological activity of 12-carboxy-JA, as well as of JA-Ile-glucosyl ester and JA-Ile methyl ester are unknown (modified after Reference [132] with permission).

JA can be converted into active, partially active and inactive compounds. At least twelve metabolic pathways converting JA or a derivative formed from JA are known so far. Among them are conjugation with amino acids, hydroxylation, carboxylation, decarboxylation, methylation, esterification, sulfation, *O*-glycosylation, and lactone formation of 12-OH-JA derivatives (Figure 4) [3,129,130,131].

Most of these reactions may be attributed to a balanced ratio of the different JA derivatives (cf. below). Some the derivatives are specifically active in distinct stress responses or few developmental processes such as leaf movement. Aspects on metabolism of JA compounds have been discussed in the following recent reviews: References [1,8,9,132,133]. 

### 8.1. Conjugation

The (*3R*,*7S*) diastereoisomer of JA-Ile was identified as the most biologically active JA compound [134]. Consequently, JA perception takes only upon formation of JA-Ile which is catalyzed by jasmonoyl-isoleucine synthetase (JAR1), a member of the *GH3* gene family [135]. All members of the GH3 protein family are acyl acid-amido synthetases. Auxin, JA or benzoate can be the substrates [136]. Among them, AtGH3.11 is specific for JA [135], whereas AtGH3.15 is specific for the auxin precursor indole-3-butyric acid [137]. The requirement of a specific stereoisomer of JA-Ile for binding [134] was used to design a ligand-based antagonist of JA-Ile based on the coronatine structure [138] or identification of inhibitors of JAR1, such as jarin1 upon chemical screening [139]. The high biological activity of the epimer (+)-*7*-*iso*-JA-Ile became clear upon crystallization of JAR1 [140]. Wounding leads to dominant accumulation of (+)-*7-iso*-JA-Ile, but tomato *JAR1*-RNAi lines were downregulated only by 50–75% [141]. Possibly, other JA-conjugating enzyme(s) exist.

In the wound response, in seed development as well as in floret opening and anther dehiscence, OsJAR1 is the preferentially active enzyme [142,143,144]. OsJAR1 activity is also required for photomorphogenesis with partially redundant activity to OsJAR2 [143]. The level of JA-Ile is preferentially determined by the activity of JAR1 and metabolic conversion of JA-Ile by hydroxylation, carboxylation and hydrolysis of the JA conjugates (cf. below). In addition to JA-Ile, (+)-*7-iso*-JA-Ala, (+)-*7-iso*-JA-Val. (+)-*7-iso*-JA-Leu and (+)-*7-iso*-JA Met were identified recently as bioactive JA derivatives [145]. These conjugates have distinct binding activity in the COI1-JAZ interaction assay. Similar biological activity has been found for (+)-*7-iso*-JA-Tyr and the so-called lasiojasmonates, which are constituents of phytopathogenic fungi, where esters of JA with a furanonenoyl moiety occur [146]. Here, a bioactive JA derivative seems to be generated upon cleavage of lasiojasmonates indicating that lasiojasmonates act as an inactive pool of JA compounds [18,146].

The above-mentioned gene *AtJAR1 (AtGH3.11)* was identified in a mutant screen on JA-insensitivity [135]. In a suppressor screen with the temperature sensitive mutant *constitutive photomorphogenic1* (*cop1*), the mutant *Far-red (FR) insensitive219* (*FIN219*) was identified to be affected in the same gene, *AtGH3.11* [147]. *FIN219* is an auxin-regulated gene which acts as a positive regulator of FR light signaling via direct interaction with COP1, indicating a link between light, JA and auxin signaling. JA was shown to regulate phytochrome A (phyA)-perceived FR signaling during photomorphogenesis [148] as indicated by longer hypocotyls of mutants such as *coi1*, *aos*, *jar1/fin219, phyA* under FR light compared to the wild type [149]. Correspondingly, JA-biosynthesis genes are induced by phyA [150]. The link between phyA signaling and JA signaling was further supported by identification of *FIN219* as negative regulator of shade avoidance responses [151] and by integration of JA and phyA signaling during shade avoidance responses via stability of the repressor JAZ1 [149]. JAR1/FIN219 interacts, however, with the FIN219-interacting protein (FIP1), which is a member of the glutathione S-transferase gene family. A recent analysis of the crystallized complex of FIN219 and FIP1 led to mechanistic insights into the adenylate-binding required for high-affinity mode during JA signaling [152]. These various data on the metabolic step of JA conjugation illustrate how the complex JA-Ile homeostasis is regulated by diverse environmentally and developmentally dependent processes of JA responses.

### 8.2. Hydroxylation

For a long time, direct hydroxylation of JA to 12-OH-JA has not been detected, except for a putative activity of a fungal monooxygenase [153]. Recently, a four-member gene family named *JASMONATE-INDUCED OXYGENASEs* (*JOXs*) has been found in Arabidopsis [154]. JOXs belong to the 2-oxoglutarate Fe(II)-dependent oxygenase gene family which hydroxylate plant hormones thereby attributing to their inactivation. The same gene family, but called JASMONIC ACID OXIDASES (JAOs), has been identified by another group showing activity of JAO2 in basal defense and responses to *B. cinerea* infection [155].

JA-Ile can be hydroxylated by several members of the *CYP94* gene family (*CYP94B3, CYP94C1*). More recently, additional family members such as CYP94B1 and CYP94B2 with hydroxylation activity were found [156,157]. Hydroxylation attribute to JA catabolism via the ω-oxidation pathway. Quadruple mutants revealed semi-redundant action of the family members [156]. The signal transduction pathway for JA-Ile is altered in wounded leaves or leaves infected by *Botrytis cinerea,* if *CYP94* gene family members were overexpressed or mutated [158,159]. A new balance of the active JA-Ile and its inactive hydroxylated derivative is sustained under these conditions including cleavage of JA-Ile and 12-hydroxy-JA-Ile by the amido-hydrolases ILL6 and IAR3 (reviewed in Reference [129]). Interestingly, the JA-Ile homeostasis is sustained by different metabolic reactions during leaf injury, infection by *B. cinerea* and during development of flowers [129].

Triple mutants of *CYP94B1*, *CYP94B3* and *CYP94C1* revealed accumulation of JA-Ile without stronger wound response. In contrast, they showed symptoms of JA-Ile deficiency and normal responses to exogenous JA which is difficult to understand [160]. 

The amido-hydrolases ILL6 and IAR3 regulate simultaneously JA and auxin homeostasis which indicates a cross-talk between JA and auxin signaling [161]. *ILL6* and *IAR3*-overexpressing lines have a JA-Ile deficient phenotype suggesting that they are not involved in a futile cycle of hydrolysis versus JAR1-mediated re-synthesis [161]. The amido-hydrolases are active in the endoplasmic reticulum, where the CYP94s are located [157,162]. JIH1, a homolog of AtIAR3 in *Nicotiana attenuata*, regulates also the JA-Ile level [163] and *N. attenuata* CYP94B3 contributes to attenuation of resistance to herbivores [164].

The cellular JA-Ile level, regulated by the balance between JAR1-mediated JA-Ile synthesis and ILL6/IAR3-mediated JA-Ile hydrolysis, might be affected by another metabolic conversion. In maize, sex determination is dependent on formation of staminate and pistillate florets from an initially bisexual floral meristem via action of the tasselseed1 (TS1)-encoding LOX indicating requirement of JA [165] and the counteraction of the silkless1 (SK1)-encoding gene. *SK1* has been shown to encode an UDP-glucosyltransferase, and constitutive expression of *SK1* led to depletion of JA and OPDA levels in tassels [166]. This suggests that the LOX/JAR1-mediated JA-Ile formation in tassels is attenuated by SK1-mediated glucosylation of JA-Ile. Formation of glucosylated JA/JA-Ile is a late and abundantly appearing wound response [156,157,158,159] (cf. Part 8.7).

### 8.3. Carboxylation

In addition to the above-mentioned hydroxylation reaction, CYP94C1, CYP94B1 and CYP94B2 exhibit stepwise oxidation activity leading to carboxylated 12-OH-JA-Ile [156,157,158,159]. 12-carboxy-JA was recently identified as a novel JA derivative in floral tissues by a non-targeted metabolite analysis. 12-carboxy-JA is presumably formed from 12-carboxy-JA-Ile upon IAR3 activity [156].

The main result of hydroxylation and carboxylation of JA compounds is the switch off in JA and JA-Ile signaling due to sustainment of homoeostasis among the active and inactive JA derivatives [130,133,167]. In nature this homeostasis among the different JA derivatives may allow plants to respond flexible on different herbivores. Indeed, in genetic approaches with *Nicotiana attenuata*, different subsets of genes involved in resistance were activated by JA and JA-Ile, respectively [168,169].

### 8.4. Decarboxylation

Product of the decarboxylation of JA is *cis*-jasmone, which is volatile and has been detected as constituent of the flower bouquet in many flowering plants involved in attraction of insects for pollination. Wounding also leads to *cis*-jasmone formation. The compound is active in multitrophic interactions of plants with aphids and their parasitoids [170]. Gene expression upon JA treatment is different from that of treatment with *cis*-jasmone [171]. In maize, *cis*-jasmone can prime formation of volatile organic compounds [172]. In Araceae *cis*-jamone is the major component of the inflorescence scent bouquet attracting different pollinators [173]. Two pathways of *cis*-jasmone formation has been proposed: (i) decarboxylation of JA, and (ii) isomerization of *cis*-(+)-OPDA into *iso*-OPDA followed by β-oxidation to 3,7-didehydro-JA and decarboxylation [174]. In Chinese dark teas *cis*-jasmone is formed transiently in the first 3 days upon fungal fermentation and is used with volatile aldehydes as diagnostic marker compound among the volatiles [175]. In *L. theobromae* a single *cis*-jasmone biosynthesis pathway via *cis*-OPDA has been detected which excludes a decarboxylation of JA [176]. A jasmone hydroxylase has been identified recently in the pyrethrin-forming plant *Tanacetum cinerariifolium* as the key enzyme in synthesis of the alcohol moiety of pyrethrin insecticides [177]. It is a cytochrome P450 enzyme, designated as CYP71AT148.

### 8.5. Methylation and Esterification

JA-Me was the first JA compound detected as constituent of floral scent (cf. review [2]) and was assumed to be active in pollination and in all reactions, where JA or JA-Ile are active as signals. JA-Me, however, is not active per se. Clear evidences by transgenic approaches have shown that JA-Me has to be cleaved by esterases followed by subsequent JAR1-catalyzed conversion to JA-Ile [178]. This corresponds to the inactivity of the JA-Ile-receptor complex to bind JA-Me [179].

JA-Me transferases have been cloned from many plant species including Arabidopsis, tomato, rice, grapevine, and strawberry. Recently, the *SABATH* gene family has been characterized from Norway spruce (*Picea abies*) [180]. Among the ten gene products are methyltransferases with different specificity for indole-3-acetic acid (IAA), gibberellin (GA), salicylic acid (SA) and JA. PaSABTH4, PaSABTH5, PaSABTH10 converted preferentially JA with different biochemical properties. Phylogenetic analysis suggested that SA and JA methyltransferases have been independently evolved in gymnosperms and angiosperms [180].

Expression of the gene encoding tomato JA-Me transferase was downregulated in both the wild relative *Solanum pennellii* and the cultivated *S. lycopersicum* by drought stress, whereas JA-biosynthesis genes such as *LOX* and *AOS* were up-regulated suggesting involvement of JA in drought stress tolerance [181]. In the fungus *L. theobromae* (+)-*7-iso*-JA was isolated for the first time [15] (cf. Part 2). Now, several JA esters, so-called lasiojasmonates, have been identified in the grapevine pathogens *L. sp.* [17]. Initially, these new compounds were considered to be inactive due to data recorded with different phytotoxic and zootoxic assays [17]. However, bioactivity including perception via the JA-JAZ co-receptor complex has now been shown [18] (cf. Part 9.2).

### 8.6. Sulfation

21 sulfotransferases (STs) occur in Arabidopsis and have been comprehensively described in respect to composition, occurrence, substrate specificity and function [182,183]. One of them—AtST2a (AtSOT15)—uses specifically 12-OH-JA as substrate [184]. 3′-phosphoadenosine 5′-phosphosulfate (PAPS) is the sulfuryl group donor. Consequently, 12-HSO_4_-JA levels are altered in kinase mutants involved in the PAPS formation [185].

### 8.7. O-Glycosylation

Many secondary compounds, as well as hormones, are modified by glycosylation, preferentially glucosylation. In potato, the main substrate for glucosylation of a JA derivative is 12-OH-JA (tuberonic acid). 12-OH-JA and its glucosyl derivative were shown to have tuber-inducing properties (cf. review in Reference [3]). This is, however, an indirect role, since strong evidences support a tuber induction via leaf generated mRNAs and two TFs which activate the temperature-dependent GA formation within the stolon [3,186]. Upon wounding of leaves of tomato or Arabidopsis, 12-*O*-glucosyl-JA accumulates abundantly after JA, JA-Ile, JA-OH, JA-COOH [167,187]. In filaments of *Zea mays* or leaves of *Glycine max* 12-*O*-glucosyl-JA accumulates up to two to three orders of magnitude higher levels than that of JA [167]. A leaf closing activity in motor cells of Albizzia and Samanea species has been detected for 12-*O*-glucosyl-JA [188]. This takes place with high structural specificity of 12-*O*-glucosyl-JA [26,188,189] as well as in respect to the glucoside moiety [190].

The inactive 12-OH-JA-Ile, formed by ω-hydroxylation of JA-Ile, can form active JA-Ile-Lactone indicating conversion of an inactive JA derivative into an active one (Figure 4) [191]. Subsequently, macrolactones of 12-OH-JA-Ile were synthesized exhibiting biological activity as (*3R,7R*) and as (*3S,7S*) forms [191].

## 9. Perception, Signaling and Expression in JA/JA-Ile Dependent Processes

About three decades ago, JA-induced proteins of barley, JA-induced proteinase inhibitors of tomato, and JA-induced enzymes in alkaloid and glucosinolate biosynthesis of *Eschscholzia californica* were the first detected JA-induced gene expression programs (cf. Reference [2]). In these early days of JA research, it was common to describe how the numerous, obviously JA-related environmental cues of biotic and abiotic origin, and the numerous developmental processes led to expression of distinct sets genes involved in stress responses and development. In the last decade, many components have been identified and characterized which are involved in perception of environmental signals, in the core signaling pathway including distinct repressors and activators of gene expression, in the signal integration and finally in the integrated responses to stress and development [8,9].

### 9.1. COI1: The Critical Component of the Complex Perceiving JA-Ile

After identification of the *coronatine insensitive1* (*coi1*) mutant affected in the F-box protein COI1 [192], much effort has been put on identification of COI1 as the JA-Ile receptor. Binding experiments of labeled JA-Ile or its molecular mimic coronatine with purified COI1 protein were unsuccessful. Only upon identification of the JASMONATE ZIM DOMAIN (JAZ) proteins in 2007, direct binding of labeled coronatine to the COI1-JAZ complex could be shown [193]. Molecular docking simulation and biochemical interaction studies [194] followed by crystallization of the COI1-coronatine-JAZ complex [179] led to an indication on receptor function of this complex. Most recently, biochemical approaches revealed that JA is dynamically perceived by a COI1-JA-JAZ ternary complex. These data suggest that the primary perception of the active JA derivative JA-Ile takes place by COI1 followed by subsequent recruitment of JAZ proteins [194]. Interestingly, inositol pentakiphosphate (InsP_5_) is required for COI1 function [179,195].

### 9.2. The Core Complex in JA-Induced Gene Expression

**Figure 5:**
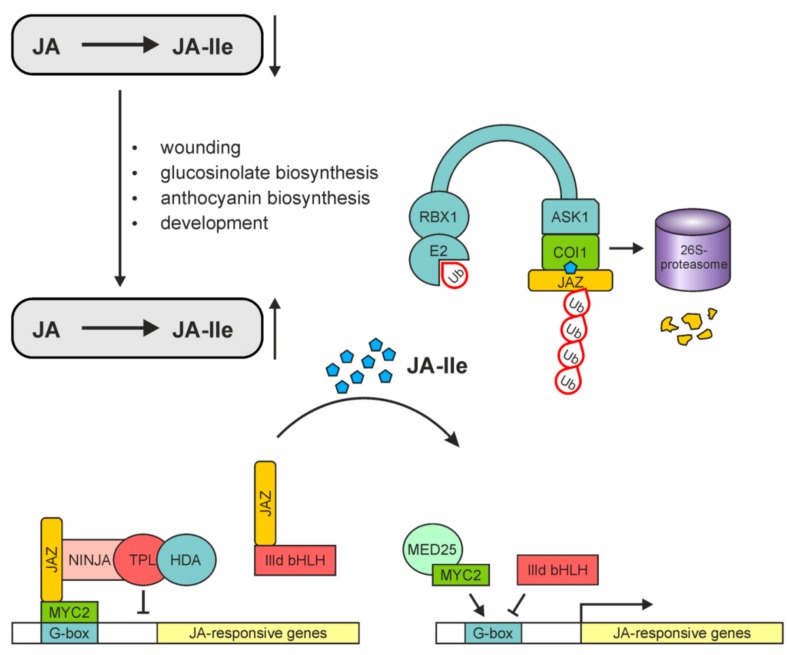
A simplified model of JA-Ile perception and signaling via the SCF^COI1^-JAZ co-receptor complex. At low level of JA-Ile, the JAZ repressors recruit the co-repressor complex consisting of NINJA, TOPLESS (TPL) and the histone deacetylase (HDA), interact with and repress positive regulators of JA signaling such as the transcription activator MYC2, and also inhibits negative regulators of JA signaling such as the transcriptional repressors IIId bHLH factors. Upon external stimuli or developmental changes levels of JA-Ile are elevated leading to perception of JA-Ile by the COI1-JAZ co-receptor and degradation of JAZs via the 26S proteasome. The downstream transcription factors are activated to regulate synergistically or antagonistically expression of JA-responsive genes and JA responses. MYC2 associates with the MED25 subunit of the mediator complex, binds to the G-box motif of the target promoters and activates JA-responsive genes. The IIId bHLH factors antagonize MYC2 via competitive binding to the G-box motif and inhibiting JA-responsive genes. COI1, ASK2, CULLIN1, Rbx and E2 are components of the SCF^COI1^ complex. Ub, ubiquitin. Modified with permission from Reference [3] (color in print).

JA-Ile-mediated promotion of SCF^COI1^-JAZ-co-receptor complex formation [193,196,197] and activity of JA-Ile as the ligand of the complex [179,194] are important in jasmonate perception. This is the basic module for JA-Ile-induced gene expression. In the last couple of years, co-receptor function of JAZ was assumed by data on binding of JA-Ile at the interacting COI1 and JAZ proteins [198,199]. The basic scenario of JA-Ile-mediated gene expression is a balanced activity of repressors and activators among the TFs (Figure 5).

In the absence of JA-Ile, JAZs repress the switch on in gene expression by activators such as MYC2 bound to JA-Ile-responsive elements of promoters in JA-Ile-inducible genes. Any increase of JA-Ile levels will lead to proteasomal degradation of the JAZ proteins thereby allowing activity of MYC2 to regulate positively gene expression. Due to competition for binding among the repressors and activators, a fine tuning of gene expression will be sustained (for details cf. References [6,9]). As mentioned above, in *M. polymorpha* essential components of JA signaling such as repressors, transcription factors, or COI1 homologs were found, even though no JA occurs [11], suggesting occurrence of a so-far-unidentified ligand for perception. Dinor-*cis*-OPDA and dinor-*iso*-OPDA have been identified recently to be these ligands in COI1-mediated signaling of *M. polymorpha*. This points to its functional conservation and co-evolution of a complex biosynthetic pathway of JA and receptor specificity [26].

In all higher plants, however, the numerous JA responses under different stress conditions and developmental processes are regulated by a homeostasis of regulatory modules containing repressors, activators, the ligand JA-Ile and the SCF^COI1^-JAZ-co-receptor complex (Figure 5). Even though most signaling pathways contain COI1, some pathways are COI1-independent. All these aspects of JA-perception and JA-induced gene expression could be only briefly reviewed here. For further details, including environmental stress responses and development, see References [6,9,200].

## Abbreviations

α-LeA	α-Linolenic Acid
13-HPOT	(13S)-Hydroperoxyoctadecatrienoic Acid
*cis*-(+)-OPDA	*cis*-(+)-12-Oxophytodienoic Acid
OPDA-Ile	*cis*-(+)-12-Oxophytodienoic Acid Isoleucine Conjugate
JA	Jasmonic Acid
JA-Ile	Jasmonic Acid Isoleucine Conjugate
OPC-8	3-Oxo-2-(2-Pentenyl)-Cyclopentane-1-Octanoic Acid
*acx1*	Acyl CoA-Oxidase1
AOC	Allene Oxide Cyclase
AOS	Allene Oxide Synthase
*coi1*	Coronatine Insensitive1
*dad1*	Delayed Anther Dehiscence1
*jai1*	Jasmonic Acid Insensitive1
JAR1	JA-Amino Acid Synthetase
JAT1	Jasmonic Acid Transporter1
13-LOX	13-Lipoxygenase
*myc2*	bHLHzip Transcription Factor MYC2
OPR3	OPDA Reductase3
PLA1	Phospholipase A1
PLIP2,3	Plastid Lipase2,3
